# The Future of NMR Metabolomics in Cancer Therapy: Towards Personalizing Treatment and Developing Targeted Drugs?

**DOI:** 10.3390/metabo3020373

**Published:** 2013-05-17

**Authors:** Marie S.A. Palmnas, Hans J Vogel

**Affiliations:** Departments of Biological Sciences and Biochemistry and Molecular Biology, University of Calgary, Calgary Alberta T2N 1N4, Canada; E-Mail: msapalmn@ucalgary.ca

**Keywords:** NMR, metabolomics, cancer, personalized medicine, drug toxicity, targeted drugs

## Abstract

There has been a recent shift in how cancers are defined, where tumors are no longer simply classified by their tissue origin, but also by their molecular characteristics. Furthermore, personalized medicine has become a popular term and it could start to play an important role in future medical care. However, today, a “one size fits all” approach is still the most common form of cancer treatment. In this mini-review paper, we report on the role of nuclear magnetic resonance (NMR) metabolomics in drug development and in personalized medicine. NMR spectroscopy has successfully been used to evaluate current and potential therapies, both single-agents and combination therapies, to analyze toxicology, optimal dose, resistance, sensitivity, and biological mechanisms. It can also provide biological insight on tumor subtypes and their different responses to drugs, and indicate which patients are most likely to experience off-target effects and predict characteristics for treatment efficacy. Identifying pre-treatment metabolic profiles that correlate to these events could significantly improve how we view and treat tumors. We also briefly discuss several targeted cancer drugs that have been studied by metabolomics. We conclude that NMR technology provides a key platform in metabolomics that is well-positioned to play a crucial role in realizing the ultimate goal of better tailored cancer medicine.

## 1. Introduction

Metabolomics, the analysis of the complete set of metabolites in a defined biological compartment, is a relatively novel approach, yet it is predicted to rapidly become a standard tool in the biotechnology and pharmacological sectors. The global metabolomics market was recently estimated to reach over 860 million US dollars by the year 2017 [1]. Metabolomics is starting to gain broader acceptance as the technology advances and as the scientific literature on the topic matures. To date, nuclear magnetic resonance (NMR) metabolomics has already been successfully applied to study various cancers such as ovarian [2], breast [3], pancreatic [4], oral [5], esophageal [6], lung [7], prostate [8], bladder [9], and colorectal malignancies [10,11]. It is clear that cancer patients present with metabolic profiles that are different from those of healthy controls and patients with benign diseases [2,4,5,6,7,9]. Moreover, the site [12], the stage [13], and the location [10] of the tumors have been shown to further alter the metabolome. It is well known that cancer cells have an altered metabolism, most often mentioned in the context of the Warburg effect [14]. However, the characteristic increase in glycolysis is of a complex nature, which was recently addressed using multiple metabolomics approaches [15,16]. The identification of specific cancer biomarkers has been predicted to be one of the catalysts that will lead to faster growth and expansion of the field. A simple blood test providing metabolomic biomarkers would be considerably cheaper than genome sequencing, or a complete proteome analysis, but it may be able to fulfill the same aims: earlier detection of cancer and to provide information that can aid in the choice of an optimal cancer treatment. To date, there are no FDA approved metabolomics tests for cancer, however metabolomics is currently being used by the FDA for biomarker discovery [17].

There has been a recent shift in how cancers are being viewed and treated. Currently, tumors are defined not only by where they are located (e.g., colon, breast, and brain) but also by their molecular characteristics. The presence of mutations affecting hormone receptors and oncogenes, such as the HER-2 receptors in breast, and K-RAS in colorectal tumors, has started to play a part when determining treatment plans [18,19]. However, the majority of cancers currently lack such markers and the present markers are far from absolute. There is considerable heterogeneity within the current definitions, exemplified by the fact that patients who are given an identical diagnosis react differently to the same therapy and have different outcomes. Important cancer subtypes are currently ignored by routine protocols but the tumor heterogeneity is being addressed with the development of targeted therapeutics. These drugs represent a new type of therapeutic agents that are designed to provide increased tumor specificity, higher efficiency, and less side effects [20]. Metabolomics can facilitate the shift from the dominating “one-size fits all” approach to a more tailored type of cancer medicine by identifying subgroups of patients that will benefit from a specific drug, as well as by identifying patients that are likely to experience toxicity or develop resistance. Metabolomics tools can identify new biological targets, find new use for drugs already on the market, and can further be applied in drug development studies to provide insight into the biological mechanisms of action of the drug and its off-target effects. For example, NMR metabolomics has already been used to evaluate the efficiency of both radiation and chemotherapy [21,22].

NMR spectroscopy is one of the three main methods currently being used for metabolomics studies, alongside gas chromatography and liquid chromatography coupled with mass spectrometry (GC-MS, LC-MS). In this systematic review we discuss the role of NMR metabolomics in drug development and personalized medicine, as we present what has been reported to date and touch on some basic aspects of NMR metabolomics. Since, this review forms part of a special issue “NMR-based Metabolomics and Its Application”, MS-based studies will not be discussed, even though they have also made important contributions to the field of cancer metabolomics. Here, we aim to provide metabolomics researchers and cancer researchers with insight in the advantages of NMR metabolomics as we survey the current status of the field.

### 1.1. Nuclear Magnetic Resonance Spectroscopy

Metabolic fluxes are highly dynamic, changing according to a broad range of factors. The complexity and constant changes require consistency in the measurements, in order to avoid additional variance (for further discussion, see below). NMR spectroscopy is a highly reproducible tool and currently one of the major players in the field of metabolomics. The samples are never in direct contact with the equipment, resulting in minimal contamination between samples. Analytical variation is considerably lower than the biological variation, as highlighted by the multi-site COMET study by Lindon *et al.* [23]. Each sample can, moreover, be re-run with only minor changes in results. Another part of the reproducibility lies in the minimal requirements for sample preparation. For biofluids, samples are close to their native state and variability due to sample preparation is kept to a minimum. Urine is easily obtainable, inexpensive, and currently one of the most commonly used biofluids in metabolomics studies [24]. Despite having low protein content, it produces complex spectra and many peaks are left unidentified. Metabolic profiles of serum and plasma are easier to interpret and they are almost fully assigned, but this will often require removal of larger molecules such as proteins [25,26,27]. Native serum can also be used, and provides valuable information on the content of lipids and lipoproteins [28]. However, the presence of these high molecular weight biomolecules results in broad signals that overlap with the peaks for the smaller metabolites. While it is possible to identify the metabolites in such spectra, filtration is needed, in our experience, for reliable and reproducible quantification. A 3-kDa micro centrifuge filter will usually remove all lipids and proteins, resulting in NMR spectra with flat baselines and well-defined, quantifiable peaks of the small molecule metabolites, as has been described [29]. Such spectra can be quantitatively analyzed using a database of known metabolite spectra, such as those found in the Chenomx software for example [30]. The broad protein and lipid particle peaks in NMR spectra of serum and plasma can also be removed during spectral acquisition by the application of special NMR pulse sequences such as the Carr-Purcell-Meiboom-Gill (CPMG) spin-echo experiment [31,32]. However, such experiments can alter the spectral intensities measured for metabolites and hence they need to be used judiciously. In addition, J-resolved 2D NMR spectra can be deployed to provide improved spectral resolution in crowded regions of serum, plasma, or urine ^1^H NMR spectra [33,34,35,36], but these experiments have yet to find wide applications in large scale clinical metabolomics studies.

NMR detects molecules based on their magnetic properties. Multiple nuclei can be used for NMR analysis, including ^1^H, ^31^P, and ^19^F with a natural abundance of 100% and isotopes such as ^13^C and ^15^N that require stable isotope labeling, because of their low natural abundance (1.1% and 0.4% respectively). ^1^H NMR is the most predominant in the field and it produces high-resolution spectra with good sensitivity because of its inherent high sensitivity and its high abundance in biological systems. In this review, the main focus will be on the applications of ^1^H NMR metabolomics. ^1^H NMR will henceforth simply be referred to as NMR. It is interesting to note that in a clinical context the term magnetic resonance spectroscopy (MRS) is often used interchangeably with NMR, mostly to avoid the use of the word *nuclear*, which can introduce concerns for those unfamiliar with the principles of the technique.

The NMR instrumentation has undergone major improvements in recent years. High-field, ultra-shielded magnets equipped with cryoprobes are now available, offering higher sensitivity and smaller magnetic stray fields. The latter is important as it makes it easier to introduce such modern instruments in hospital settings. Biofluids, such as the aforementioned serum, plasma, and urine, are most commonly used for analysis, but the use of cerebrospinal fluid, saliva, synovial fluid, and fecal water has also been reported in a clinical context [37,38,39,40]. For studies of intact tissues (e.g., biopsy material), high-resolution magic angle spinning (HR-MAS) can be used to achieve spectra with a quality that is comparable to those of extracted molecules [41]. As the name indicates, in HR-MAS the higher resolution is achieved by spinning the samples at a specific angle (“the magic angle”) [41]. In a regular high resolution NMR experiment, tissues would give rise to broad peaks, with increased overlap of signals and with peaks disappearing into the baseline. HR-MAS also makes it possible to avoid extraction procedures that otherwise would effect the metabolic composition. On the other hand, changes in the metabolites are hard to avoid during the HR-MAS experiment, as the enzymes in the tissues are still active.

Using samples from patient-based metabolomics studies is likely to be more representative of the processes occurring in the human body, when compared to the analysis of cultured cell lines. However, even far progressed tumors rarely constitute more than 1% of the total body weight; consequently it is unlikely that all changes observed in body fluids are due to the cancer itself, as there will also be contributions from the immune response. Cell lines are less influenced by external factors and are in some cases also preferable when testing new drugs or drug combinations. Such studies may thus be a crucial first step in drug development and personalized cancer medicine, but the results should eventually be validated in studies of cancer patients.

### 1.2. NMR Metabolomics

NMR is a well-established tool in protein structural, protein ligand binding, and protein-protein interaction studies [42,43,44]. It is the foundation for structure-based drug design studies and it has been used to provide insight into drug-target interactions [45,46]. In recent years NMR has been utilized to analyze the metabolome. The human metabolome represents the ongoing processes in our body, including homeostasis maintenance such as energy metabolism and dynamic fluxes such as response to disease. It is highly influenced by various factors such as diet, gender, and age, as well as body composition [47] and intake of drugs, as discussed below. Because of its high sensitivity and rapid response to changes in the environment, the metabolome is often claimed to best reflect the phenotype, when compared to other “omes” such as the genome and the proteome [48]. The complexity requires reliable and reproducible experimental tools as well as carefully thought-out study designs to obtain meaningful results.

The rapid changes within the metabolome in response to therapeutic agents and disease are an advantage. The presence of disease or a toxic response can often be detected before clinical symptoms manifest, offering possibilities for earlier diagnosis or the prevention of side effects. Drug response and disease progression can, moreover, be monitored over time in longitudinal studies. Metabolomics is objective in its nature and non- or minimally invasive when using biofluids. The main drawback of NMR is the relatively low sensitivity, necessitating a minimum abundance of metabolites in the micromolar range and thus larger sample sizes are required than for mass spectrometry. While GC-MS and LC-MS offer higher sensitivity, issues concerning peak identification, quantitation, and reproducibility have not yet been fully addressed. The capabilities and limitations of GC-MS are discussed in a recent review by Koek *et al.*, who also propose changes on how to optimize experimental procedures, validation and data processing and analysis [49]. The potential of NMR and MS metabolomics were rapidly identified by Griffin and Shockcor as providing a useful approach to study cancer cells, and by Nicholson and colleagues to evaluate drug toxicity [50,51].

A complete listing of metabolomics approaches is presented in the review paper by Gowda *et al.* who also report on the types of cancers that have been investigated [52]. For an overview of the applications of NMR metabolomics and the important part it can play in tumor characterization the reader should consult the article by Bathen and colleagues [53]. A recent review on systems biology by Dunn *et al.* can also be consulted, which describes the use of NMR and MS to evaluate the effects and health aspects of diet and drugs in mammals [54]. In the following, it is important to note that this review relies on the biological interpretations presented by the authors of the articles herein cited. Consequently, we encourage the reader to refer to the original articles for specific results and discussions of their respective biological impact.

### 1.3. Targeted Therapeutics

The group of targeted cancer therapeutics includes agents with widely different molecular characteristics. Small-molecule drugs and monoclonal antibodies dominate over the less common proteasome inhibitors and retinoids. Targeted cancer drugs can furthermore be grouped according to their molecular targets. FDA approved inhibitors of signal transduction pathways, growth factor receptors, and apoptosis have been studied by metabolomics approaches, as presented in Table 1 and as described briefly below. One of the studies applied MS metabolomics and will not be discussed [55]. Approved drugs belonging to the other drug classes, consisting of anti-angiogenic agents, monoclonal antibodies, promoters of the immune system and regulators of gene expression and other cellular functions have to the best of our knowledge not yet been studied by metabolomics approaches. A complete listing of all FDA approved targeted cancer therapeutics can be found on the homepage of the National Cancer Institute [20]. Metabolomics studies investigating potential new agents will be discussed in the below Section 2.

Tamoxifen citrate (Nolevadex) is one of the best known cancer therapeutics, which has been administered to treat tumors of the breast since 1977 [56]. It has been approved to reduce breast cancer incidence in the high-risk population, to prevent recurrence and invasiveness of breast tumors, and to increase survival in ER-positive patients [56]. NMR metabolomics has been used to study the impact on the liver, after long-term expose to Tamoxifen [57]. In this study, 344 female Fisher rats were given the estrogen antagonist Tamoxifen, the synthetic estrogen Mestranol or Phenobarbital, acting on the central nervous system, for six months, after which they were sacrificed and their livers were removed. Both polar and lipid extracts from the livers were analyzed, showing higher concentrations of fatty acids in the Tamoxifen treated animals. Higher levels of succinate, acetate, and formate were detected in both Tamoxifen and Mestranol treated rats. The authors concluded that these changes likely were due to the estrogen receptor (ER)-activity of the two drugs [57].

Tamoxifen is also approved to treat breast tumors to avoid metastasis [56]. Metastatic breast cancers are difficult to diagnose and invasive biopsies are often needed for clinical evaluation. In a recent pilot study, sera from over 500 patients with metastatic breast cancers were analyzed to investigate the potential of Lapatinib treatment [58]. Serum was collected pre-treatment and at multiple times during treatment with Placitaxel, which was administered in combination with either Lapatinib or placebo. No correlation could be found between the metabolic profiles and toxicity or other outcomes, when all patients were included or when intra-individual comparisons were made. However, for HER-2 positive patients given the combination treatment, overall survival (OS) and time to progression (TTP), marking the time between randomization and progression of breast cancer or death, could be predicted by the metabolic profiles of sera taken at nine weeks. Larger TTP values were correlated to lower concentrations of phenylalanine and glutamate and higher concentrations of glucose. The authors suggested that patients with higher sensitivity to Lapatinib and Paclitaxel treatment may be identified in the future by using an NMR metabolomics approach [58]. However, these findings need further validation in order to draw more detailed biological conclusions and to realize such a test.

Imatinib (Gleevec) is considered one of the biggest successes in the area of targeted cancer drugs. The agent targets the receptor tyrosine kinase of the BCR-ABL fusion protein, that is present in the majority of cases of chronic myeloid leukemia [59]. Several follow-up studies have been conducted since the first clinical trial was performed in 1998, supporting the initial promising results [60,61,62]. A complete cytogenic response in up to 87% of patients has been reported, with 5- and 6-year overall survival rates of 89% and 88% respectively and with no additional side effects [60,61]. The drug is also used to treat gastrointestinal stromal tumors. The metabolome of Gleevec resistant cells was studied by Dewar et al and will be further described below [63].

Lastly, Bortezomib (Velcade) is a proteasome inhibitor that is used as second or third-line therapy for multiple myeloma and mantle cell lymphoma. Its synergistic effects with Belinostat, investigated by NMR metabolomics, will be described further below.

**Table 1:** FDA approved targeted cancer therapeutic agents investigated by metabolomic approaches.

Class	Type	Drug	Cancer	Reference
Estrogen Receptor Inhibitors	Selective estrogen receptor modulator (SERM)	Tamoxifen citrate (Nolvadex^®^)	Ductal carcinoma *in situ* (DCIS) post radiation and surgery	[56,57]
Signal transduction inhibitors	Small-molecule drug	Imatinib mesylate (Gleevec^®^)	Multiple tumors and disorders *	[64]
	Small-molecule drug	Lapatinib (Tykerb^®^)	HER-2-/hormone-positive advanced breast cancer	[58]
	Small-molecule drug	Vandetanib (Caprelsa^®^)	Unresectable and advanced medullary thyroid cancer	[55]
Apoptosis inducers	Proteasome inhibitor	Bortezomib (Velcade^®^)	Multiple myeloma and mantle cell lymphoma (post first-line therapy)	[65]

* Gastrointestinal stromal tumors, Philadelphia chromosome positive acute lymphoblastic leukemia (Recurrent or refractory) and chronic myelogenous leukemia, chronic eosinophilic leukemia or hypereosinophilic syndrome, systemic mastocytosis, dermatofibrosarcoma protuberans and myelodysplastic/myeloproliferative disorders.

### 1.4. Drug Discovery and Development

There is a continuous drive to develop more targeted drugs. However, drug discovery is an expensive and time-consuming process. Up to 10,000 compounds might be originally investigated in order to develop one single drug, a process that typically takes up to 15 years [66]. Any development of side effects will slow down the process and every drug that fails will add to the overall cost of drug development. Hence, there is a need for tools that can provide mechanistic information about putative new drugs and their potential off-target effects. Unraveling the biological mechanism of a drug can, moreover, provide some direction as to how a drug or its use can be improved. As previously mentioned, the identification of tumor specific metabolic patterns can lead to new molecular targets. Metabolomics approaches can be used to evaluate drugs that are already in use. By having a deeper understanding of how a drug works, we can identify new combinations of drugs that have higher potency and/or lower toxicity as well as identifying diseases that may respond to an unconventional drug. Such a strategy could lead to new and better use of the drugs that have already been developed.

## 2. Investigating Existing and Potential Anti-Cancer Agents

### 2.1. Evaluating Toxicity

Amongst the very first applications of metabolomics were investigations of toxic effects of drugs and studies of their potential mechanism of action in animal models [67,68]. In the field of oncology, avoiding toxic effects is crucial, as drug toxicity presents itself as a serious limitation of the efficacy of chemotherapy [69]. Adverse side effects appear when drugs become distributed to other, healthy, parts of the body where they exert a negative impact. This results in patient suffering, a lower quality of life, and possibly in severe cases termination of the treatment. To limit toxic effects, lower doses are often used resulting in sub-optimal results [70]. Another way to decrease side effects is to make a drug more specific, so that the effect is exhibited primarily within the tumor. Such higher tumor-specificity can be achieved by using locoregional drugs, or prodrugs, that only become active once on site. ^13^C and ^1^H NMR were used by Sorg et al to evaluate the anticancer potential of prodrugs of glycoconjugated agents [71]. In this study, NMR spectroscopy was primarily used to assign the structure of the newly synthesized drugs.

**Figure 1:**
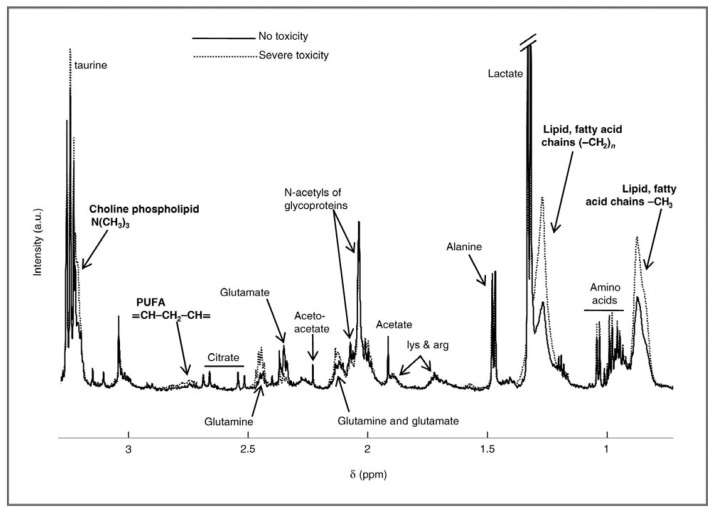
Overlay Nuclear Magnetic Resonance (NMR) spectrum of patient groups experiencing no toxic effects and severe toxicity respectively. Each line represents the mean of the group. Taken from reference [73] with permission.

Naser-Hijazi *et al.* used ^19^F to quantify the prodrug 5-fluoro-2'-deozyuridine and its metabolites in hepatocellular sarcoma tissue after exposure to the fluorine-containing cytotoxic agent [72]. Optimal dose and appropriate infusion times were determined based on the level of the degradation products as well as the tumor volume. Interestingly, low levels of the end product, α-fluoro-β-alanine, were found in the tumor. This finding was interpreted as an indication of the drugs release from the liver and reentering into the blood stream and the tumor cells [72]. Capecitabine, another prodrug of 5-fluorouracil, was evaluated by Backshall and colleagues in order to assess the cytotoxicity in patients with inoperable colorectal tumors [73]. The primary aim of this study was to identify subpopulations with a pre-disposition for side effects. Toxicity was predicted by connecting pre-treatment metabolic profiles to toxic events that were experienced post-treatment. The high-toxicity group was characterized by higher concentrations of choline containing phospholipids, polyunsaturated fatty acids, and other lipids of low-density lipoproteins, whereas tyrosine and an unassigned peak at 7.2–7.3 ppm were found to be higher in the group that experienced no or low toxicity (Figure 1). It was proposed that the extra lipids bind and interfere with proteins involved in drug metabolism, when present at higher concentrations. However, it was also stated that the lipid profile might also be reflective of an ongoing inflammatory response, hence further experiments are needed in order to confirm the proposed mechanisms. The changes in the metabolic profiles appeared to be gradual, allowing for the study of the onset and the progression of Capecitabine toxicity. Weight but not BMI, age, or gender was identified as a confounder for toxicity grade. Targeting the lipidome pre-treatment was suggested to be one way to reduce the level of toxicity [73].

Analyzing multiple biofluids [29], or using multiple metabolomics approaches, will likely provide more reliable results, validating one set of data with the other. Metabolomics approaches can in the same manner also be used alongside other targeted or untargeted “omics” techniques, acting in a complementary fashion. A growing area of importance within the “omics” and systems biology field is combining metabolomics with genome wide association studies (mGWAS). In such work one attempts to connect metabolic patterns in responders and non-responders to specific genetic traits, as recently reviewed [74]. Another example of combining methods of different nature is presented in an article by Wang *et al.* using urine, plasma and liver samples [75]. In this study, NMR spectroscopy, histopathological assessments, and current biomarkers were used of to investigate the unknown mechanism of liver toxicity caused by the anti-angiogenic drug Z24. Time-dependent changes were observed in the metabolomics data, providing information about the initial response, progression and recovery after Z24 treatment. The most pronounced changes were seen in urine with increased levels of citrate, succinate, acetate, and 2-oxo-glutamate and decreased levels of trimethylamine-N-oxide and creatinine. Plasma and liver levels of glucose and choline compounds were decreased post Z24 treatment. Polar and lipid fractions of the liver extracts showed higher levels of lactate and glutamine and triglycerides respectively. These observations led the authors to propose that the toxic response might result from the impairment of mitochondrial functions, ultimately resulting in cell death [75].

It is important to acknowledge that the overall aim of the therapy may potentially affect the choice of the dosage and treatment. If the aim is to cure, more pronounced adverse effects are usually tolerated in order to achieve the treatment goal. However, if the aim is to provide palliative care, only lower levels of adverse effects and patient suffering will be accepted.

### 2.2. Evaluating Resistance and Sensitivity

**Figure 2:**
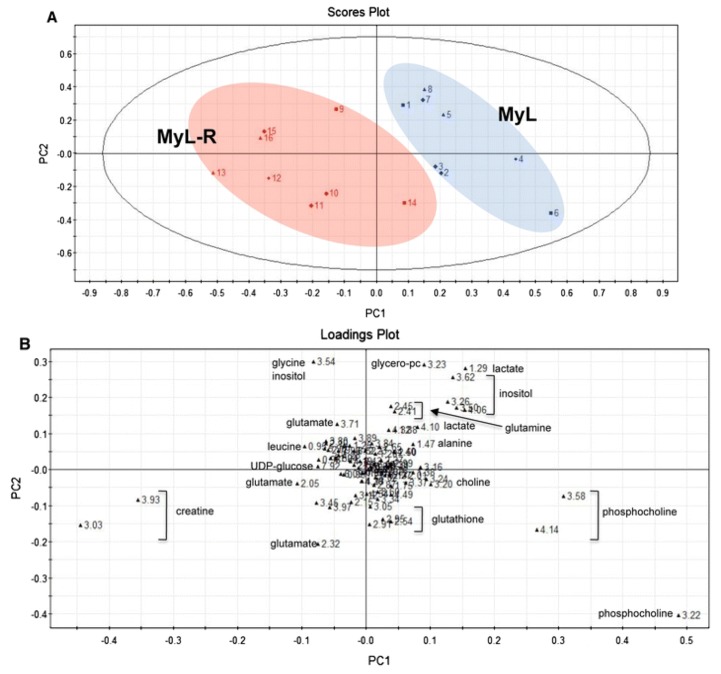
(**A**) PCA scatter plot of spectral data from normal (MyL) and Gleevec resistant (MyL-R) cells and (**B**) its loadings plot showing metabolic correlations.

As previously mentioned, Gleevec represents one of the major recent advances in targeted cancer therapies. However, as noted for many other anti-cancer drugs, some cells develop resistance against the treatment. Dewar et al studied and compared the metabolic profiles of two chronic myelogenous cell lines, with one cell line showing increased tolerance to Gleevec after having been exposed to the drug over time [63]. Both cell extracts (“fingerprints”) and cell culture media (“footprints”) were studied. The most pronounced detectable differences between the two cell lines were reported to be the total concentration of creatine and creatine-phosphate, with the resistant cell line showing increased levels. High performance lipid chromatography (HPLC) experiments confirmed the higher content of creatine containing compounds in resistant cells and could further identify that creatine-phosphate dominated the creatine pool, in contrast to the responding cells that presented a 1:1 ratio. The higher conversion of creatine to creatine-phosphate could also be monitored by ^31^P NMR. Glycolytic metabolites also contributed to the separation between the normal and Gleevec resistant cells, as seen in the scores plot of the unsupervised model (PCA) and its loadings plot (Figure 2) [63].

Wei *et al.* recently performed a study evaluating NMR and LC-MS metabolomics to predict the response to chemotherapy in breast cancer patients [76]. Serum samples were collected prior to treatment, consisting of chemotherapy followed by surgery. When comparing non-responders to partial and complete responders, isoleucine, glutamine and threonine (NMR) and linolenic acid (LC-MS) were detected to significantly correlate to treatment response. Unexpectedly, the outcome could not be related to any differences in ER, HER-2 or progesterone (PR) status, but larger sample sizes than the current number of patients (n = 28) would be needed to further evaluate subgroups [76]. Pre-treatment metabolic profiles of human glioma cells have also been reported to indicate sensitivity *versus* resistance to chemotherapy [77]. Being able to predict drug response would not only make it possible to offer responders the appropriate drug as first line treatment, but also to provide non-responders with an alternative treatment at an earlier stage.

### 2.3. Treatment Response to Different Drugs

The effect of a drug on cell metabolism is difficult to predict, even when the type of anti-cancerous effect of the drug is known. Triba *et al.* investigated the differences in metabolic profiles following treatment with either the chemotherapeutic agent doxorubicin, or the calcium chelating agent BP7033, the latter only having recently been shown to induce apoptosis [78]. Both treatments were found to reduce cell growth to a comparable extent in the murine melanoma cells that were used for evaluation. It was hypothesized that BP7033 was achieving apoptosis by forming calcium complexes. Interestingly, the cell growth inhibition of BP7033 was not of a calcium chelating nature. Control cells and the two treated cells formed three distinct groups in the multivariate scatter plot, indicating strong differences in the spectral data. Doxorubicin was concluded to act on neutral lipid metabolism and influenced the levels of inositol and lysine. The drug was further suggested to induce apoptosis, an event that correlated with decreased levels of acetate, glutamine, and alanine and an increased signal at 1.30 ppm, most likely corresponding to the methylene content of fatty acids, according to Triba and colleagues. BP7033 treated cells had high levels of glutamine and altered phospholipid metabolism displaying strong signals originating from glycerophosphocholine and phosphocholine. These findings were suggested to provide a foundation for future *in vivo* studies concerning drug mechanism; they also act as a reminder of how two drugs can achieve similar results (*i.e.*, growth inhibition) through different mechanisms [78].

### 2.4. Treatment Response in Different Cell Lines

**Figure 3:**
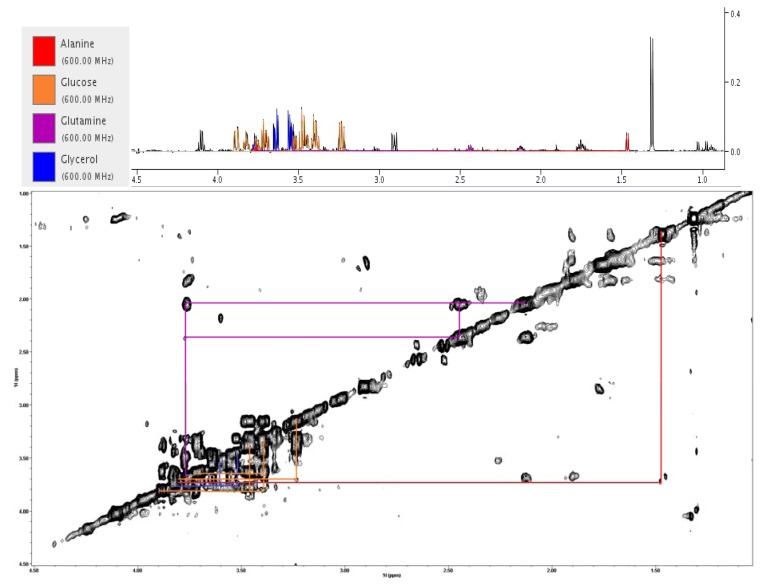
Representative ^1^H NMR spectra of region 1.0-4.5 ppm (**a**) expanded one dimensional spectrum and (**b**) the related two dimensional total correlation spectroscopy (2D TOCSY) spectrum indicating peaks and cross peaks (unpublished data).

Bayet-Robert et al applied two dimensional (2D) HR-MAS NMR to evaluate drug toxicity and drug profile in four human cancer cell lines (HepG2, 143B, MCF7, and PC3) and in non-malignant fibroblasts [79]. The cell lines were phenotyped pre-treatment and presented with common attributes such as increased levels of choline containing compounds and sulfur derivatives and decreased levels of free amino acids, such as glutamine, leucine, asparagine, alanine, arginine, and lysine (decreased in all four cell lines). The metabolic profile of the HepG2 cell line, representing the most differentiated cells, were also the most diverse with higher levels of total fatty acids, no increase in phosphocholine (PC) and with a slight increase in lactate levels. Chemotherapy resulted in few global effects, such as increased fatty acids (143B and MCF7) and an increase in PC (HepG2 and 143B), however most of the changes were cell line specific. The results highlight that the differences between cell lines are profound and will effect how one drug exhibits its effect and that this aspect needs to be considered when conducting metabolomics studies [79]. 2D A representative 2D ^1^H total correlation spectroscopy (TOCSY) spectrum can be found in Figure 3.

**Figure 4:**
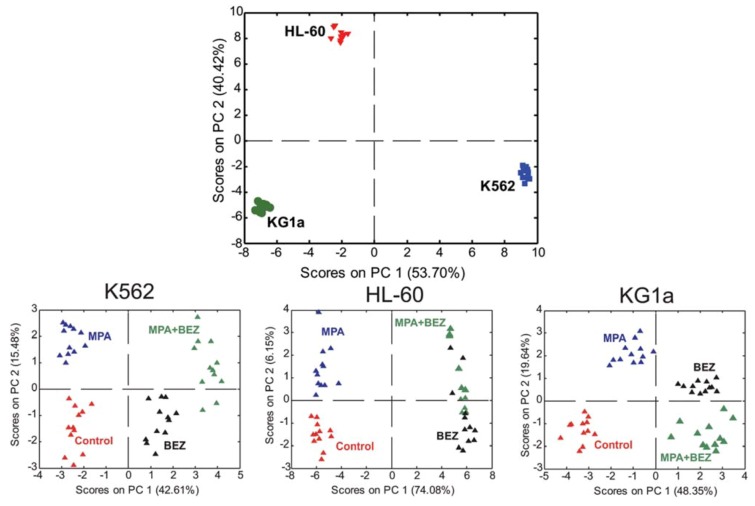
Scatter plots of unsupervised models (PCA) where every symbol represents one replicate. (**Top**) Untreated cells, showing grouping according to cell type (**Bottom**) cells post treatment of single agents Medroxyprogesterone acetate (MPA) and Bezafibrate (BEZ) and combined treatment, show grouping according to treatment. Solvent controls included. Figures adapted from reference [62] with permission.

Tiziani *et al.* treated three leukemia cell lines with Bezafibrate (BEZ) and Medroxyprogesterone (MPA), two unconventional drugs for the treatment of leukemia [80]. These two drugs are known to exhibit sub-type dependent effects, with the mechanisms of action not fully understood. One of the cell lines was chosen because it is known to respond to the combination treatment by promoting differentiation, whereas the other two cell lines had demonstrated increased apoptosis in prior studies. An increase in the level of reactive oxygen species (ROS) was observed as a global effect. Metabolic alterations could be detected after 24 hours of treatment, however these effects were inferior to the metabolic characteristics of the different cell types that produces strong clustering in an unsupervised scatter plot (Figure 4 top) supporting the results from Bayet-Robert *et al.* [79]. Interestingly, cells treated with single agents could easily be distinguished from those treated with the combinational therapy, in an unsupervised model (Figure 4 bottom). The combination of the drugs gave more pronounced effects than the agents alone, which the authors concluded to be mainly due to a stronger impact on TCA cycle intermediates and the levels of free amino acids. TCA cycle perturbations have previously been connected to ROS production and oxidative stress [81] To test this hypothesis, all three cell lines were treated with hydrogen peroxide (H_2_O_2_). A coinciding accumulation of succinate and decreased levels of alpha-ketoglutarate was observed both after combination treatment and after exposure to H_2_O_2_, supporting the notion that oxidative stress plays a major role in the mechanism of action of the drug. There were also indications of the conversion of pyruvate to malonate via acetate and oxaloacetate after drug or H_2_O_2_ treatment [80].

Hepatocellular and pancreatic cell lines were used in a similar manner to uncover the post-treatment phenotype of two anti-proliferative drugs, with completed phase II and III clinical trials respectively [65,82,83]. Belinostat and Bortezomib were investigated as combination therapy, to simultaneously inhibit histone deacetylases (HDACs) and proteasomes. Two hepatocellular and two pancreatic cell lines were evaluated post-treatment by immunoblotting and by ^1^H, ^13^C, and ^31^P NMR. HDAC inhibition was more pronounced following combination therapy than after treatment with Belinostat as a single-agent. The metabolic signature of the synergistic effect was interpreted as reflecting a decrease in proteasome activity in the form of high abundance of free amino acids and antioxidants, a response that has been coupled to decreased migration and proliferation and increased apoptosis. Both agents induced apoptosis alone, but the synergetic effect resulted in changes up to 16- and 38-fold for the pancreatic and liver cell lines respectively. The synergistic effects of the two drugs also lead to stronger anti-proliferating effects, demonstrated by Combination Index (CI) values < 1 and by achieving IC50 values at lower concentrations. Immunoblotting data was stated to provide overall support for the mechanisms of action of the two drugs. There is great need for new therapeutic strategies for liver and pancreatic tumors. Both cancers have shown resistance to standard chemotherapy and there has been very limited success in increasing the overall survival in liver cancer patients with current treatments [65]. The two drugs has previously been shown to exhibit toxic effects, leading to termination of a phase II trial for relapsed, refractory multiple myeloma [84]. Hence, these results illustrate a need for a better understanding of the synergetic effects.

The metabolic link to apoptotic response has also been investigated by Pan *et al*. [85]. In their work, two neural tumor and two glioma cell lines, were exposed to the chemotherapeutic agent cisplatin. One glioma and one neural tumor cell line were found to have higher susceptibility to the drug, resulting in nuclear condensation and fragmentation, indicating apoptosis. In responding cells, but not in non-responders, cell death was shown to be associated with increased levels of lipids, uridine diphospho-N-acetylglucosamine (UDP-GlcNAc) and uridine diphospho-N-acetylgalactosamine (UDP-GalNAc). This response was suggested to be due to an increased glucose intake, a decrease in utilization of these compounds and a mechanism to attract macrophages in order to remove the dead or dying cells. Concentrations of the glycosylated uridine metabolites and involved enzymes are known to be altered by a broad range of stresses, and has been suggested to play part in tumorigenesis and metastasis [86,87]. In this work, the two compounds UDP-GlcNAc and UDP-GalNAc were found to connect cisplatin treatment specifically to cell death in brain tumor cells [85], an observation that should be confirmed in future studies, to evaluate its clinical usefulness as a potential indicator of response.

### 2.5. Evaluating Dose Response

Dosage of a drug is coupled to the efficiency of a treatment as well as to the development of side effects. Docetaxel, a cytotoxic agent acting on microtubules, has been found to have a dose-dependent drug effect, showing perturbations in mitosis and necrosis with low doses, and increased levels of cell cycle arrest and apoptosis as the dose increases. Bayet-Robert *et al.* investigated the metabolic response induced in breast cancer (MCF7) cells after exposure to a low and a high dose [88]. As hypothesized, a dose-dependent response was observed along with variations in the levels of 40% of the identified metabolites. The cytotoxic effects of the high dose correlated to an accumulation of polyunsaturated fatty acids and a suggested increased activity of glutathione S-transferase, correlating to the depletion of the precursor glutamate. In contrast, the low dose resulted in high levels of homocysteine, which was interpreted as an indication of enzyme inhibition. Higher levels of myo-inositol, probably related to an increased production of phosphatidylinositol, were furthermore observed for cells given the lower dose. The drug response was moreover seen to consist of two phases, one initial response with higher levels of alanine, acetate and polyamines and a delayed response characterized by an increase in the levels of phospholipids. Both doses led to an accumulation of phosphocholines [88]. Clearly, these metabolomics data provided insight into the metabolic processes that were affected and the study also identified when they occurred.

### 2.6. Evaluation Unconventional Therapies

Bayet-Robert *et al*. also evaluated the potential cytotoxic effects of three marine natural products (MNP) on MCF7 breast cancer cells [89]. All three products resulted in cell death, but presented a different mechanism of action. Treatment with Kahalalide F, a compound currently undergoing phase II clinical trials, lead to swelling of the cells and to non-apoptotic cell death. Metabolomics data had in the past indicated an impact on lipid membranes, here supported by an accumulation of polyunsaturated fatty acids, phospholipids and total content of fatty acids. The pyrrole alkaloid Lamellarin D lead to accumulation of metabolites involved in the malate-aspartate shuttle, such as glutamate, aspartate, ethanol and lactate. A blockage of the electron transport chain located in the inner membrane of the mitochondria was suggested as a plausible mechanism. The third compound, Ascididemin, strongly induced apoptosis and led to an increase of gluconate, citrate, alanine, and phosphoethanolamine, which led the authors to propose a perturbation of citrate metabolism. A decrease in DNA was observed in cell lines treated by all three MNPs [89].

Metabolomics approaches have also been used to evaluate traditional Chinese medicine [90] and antibacterial agents [91]. In such traditional medicine, the scientific basis is often weak limiting its use in larger populations. By applying metabolomics and other objective techniques to study individual components, it would be possible to evaluate such compounds and potentially gain a deeper understanding of traditional and alternative medicine.

## 3. Personalizing and Stratifying Medicine

### 3.1. Survival and Outcome

**Figure 5:**
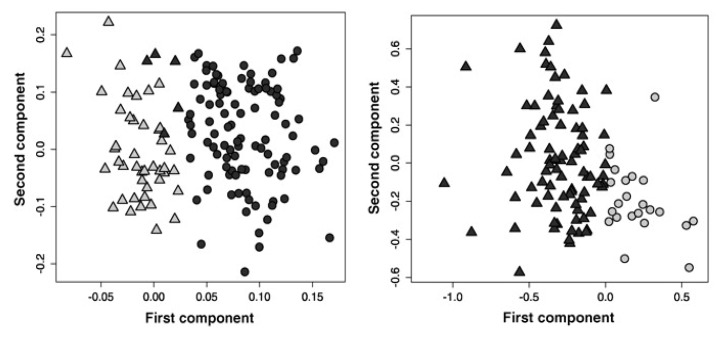
Scatter plots of PLS-DA of validation set based on training set models for (**left**) patients with metastatic colorectal cancer (dots) healthy participants (triangles) and (**right**) short overall survival (OS) group (dots) and long OS group (triangles). Items adapted [92].

Overall survival (OS) is used to determine and explain prognosis and to develop a treatment plan. For colorectal patients, OS is predicted from the presence or absence of K-RAS mutations, blood cell counts and Eastern cooperative oncology group (ECOG) performance status as well as from the serum levels of certain proteins such as lactate dehydrogenase and the metabolite bilirubin. Bertini *et al.* collected serum samples from 153 metastatic colorectal patients and 139 healthy controls [92]. For patients, blood was collected prior to the initiation of third-line treatment, consisting of a combination of Cetuximab and Irinotecan. Cancer patients and healthy controls were clearly distinguished from each other by multivariate statistical analysis of the spectral data (Figure 5a). The cancer patients presented with metabolic alterations interpreted as perturbations in energy metabolism, with variation in citrate, alanine, and pyruvate concentrations, and a more pronounced inflammatory response characterized by the N-acetyl group of glycoproteins and the –CH_2_-COOR group of lipids. The two latter metabolites were furthermore correlated to short OS. Creatine, valine, and lipid signals further contributed to the strong separation between patients with low *versus* high values of OS (Figure 5). Intriguingly, the statistical analysis did not find K-RAS or ECOG to be reliable predictors of OS, in contrast to metabolomics data and the serum marker C-reactive protein (CRP). This study was part of a phase II trial studying the effects of the Cetuximab/Irinotecan combination treatment [92].

Patient outcome after chemotherapy has also been found to correlate to the abundance of carnitine and acetyl carnitine in multiple myeloma patients [93]. In the study by Lodi *et al.*, paired serum and urine samples were used to identify metabolic patterns correlating to disease progression, which resulted in the detection of a difference in the metabolome of patients who relapsed and patients that went into remission. Current markers for multiple myeloma can predict outcome on a population level. This study aimed to identify markers useful to the individual patients [93].

### 3.2. Identifying Subtypes

Contradictory results in OS has been reported for breast cancer patients treated with the anti-angiogenic drug Bevacizumab (Avastin), which resulted in revoking the FDA approval of the drug for the treatment of breast cancer [94]. It was proposed by Borgan et al that the reported differences in drug response were due to the great diversity of tumors that had been studied, and that the utility of Bevacizumab could only be demonstrated after having identified responders from non-responders [95]. It was hypothesized that specific subtypes of breast tumors were more susceptible to the drug and that several metabolic characteristics correlating to drug response could be identified. Two xenograft models of basal- and luminal-like breast cancers were used and exposed to either chemotherapy (Doxorubicin) alone, or in combination with Bevacizumab, often used to enhance the potency of other drugs to treat several types of cancers. The basal-like breast cancer xenografts responded to the combination therapy whereas the luminal-like counter part did not, a difference partly ascribed to the adverse effects on the levels of glycerophosphocholine (GPC). The decrease in GPC in responding cells was seen both following treatment with the single agents alone and as combination therapy. The increased GPC levels in laminal-like cells were observed along with increased concentrations of phosphocholine as well as total choline and decreased levels of taurine. The same research group had previously investigated the impact on tyrosine kinases and other parts of the proteome using the same xenograft models and therapeutic agents. The results showed similar patterns with the transcriptomic data presented in this article. As hypothesized, certain subgroups of breast cancers were shown to have a higher benefit of Bevacizumab and potentially other anti-angiogenic drugs [95].

### 3.3. Case Reports

Metabolomics has been proposed as a tool to evaluate treatments on an individual level and to compare tumor and healthy specimens from the same patient. However, the literature on this topic is still very sparse. Two examples will be given to illustrate the concept, even though the methodology goes a little beyond the focus on NMR metabolomics of this mini-review. Abaffy *et al.* compared the metabolome of malignant melanoma tissue with a sample of healthy nearby skin taken from a 49-year old male [96]. The aim of the study was to identify possible biomarkers that could be used for early detection of skin cancer. Untargeted GC-MS detected nine metabolites to be elevated in the cancerous tissue, including dodecane, nonanal, 1,3,5-trimethyl benezene, and 1-hexadecanol. Moreover, 23 metabolites were present in the malignant tissue only. These included decane, undecane, 4-methyldecane, ethylene oxide, isopropanyl palmitate, and Bis (2-ethylhexyl) phthalate. The strongest candidate, 1-hexadeconol was increased 35-fold. All mentioned metabolites have previously been reported to be increased in melanoma cells [96].

Vriens *et al.* evaluated glucose metabolism and vessel perfusion and permeability in two male patients with metastatic colon cancer undergoing Bevacizumab treatment as part of their cancer therapy [97]. Measurements were taken at baseline, after three cycles of therapy and at a late stage (9-12 cycles). Dynamic contrast enhanced magnetic resonance imaging (DCE-MRI) revealed signs of normalization of tumor vasculature and a reduction in glucose turnover. Despite treatment, both patients died during the study having an OS comparable and slightly higher to the mean survival rate [97].

## 4. Discussion and Conclusions

NMR metabolomics has been used to evaluate drugs and tumor characteristics in order to help develop new agents and to maximize the beneficial effects of drugs that are currently on the market. NMR is a key method in metabolomics and the technology continues to improve, allowing NMR spectroscopy to remain involved in future drug research and development, facilitating the movement toward personalized medicine.

To this date, only a limited number of approved anticancer drugs have been investigated by metabolomics methods. However, over time we believe that metabolomics approaches will come to play a prominent role in future clinical studies when developing new drugs. Acknowledging that unique molecular characteristics will determine how a drug is tolerated and how this in turn influences the outcome of the patient is of utmost importance. Better specific molecular targeted drugs and suitable stratification of patients are two examples of how cancer treatment can become better tailored towards the tumor and patient. To be able to quickly identify the best drug (or drug combination) for a specific patient should lead to a more efficient treatment, reduced patient suffering and enhanced health-economical benefits. Furthermore, if such a first line treatment is successful, subsequent second and third-line treatments can perhaps be avoided in many cases.

In closing, it is important to reiterate that metabolomics of biofluids has the advantage of being relatively cheap and that it is relatively non-invasive or at least minimally invasive. The obtaining of blood and urine samples is already a well-accepted procedure in common clinical practice. Identifying subgroups of patients that might benefit from a certain drug by taking a simple blood sample would not only benefit the patient but would also provide health-economical advantages. In order to realize such a test, many more appropriately designed metabolomics studies are needed, investigating the metabolic differences in responders and non-responders, in patients not experiencing toxic effects *versus* those who do and lastly, to identify those groups of patients where combination therapies might be advantageous. The biology of a drug is sometimes poorly understood, which currently leads to a less than optimal usage, but NMR (as well as GC-MS and LC-MS) metabolomics can provide much better insight. In a similar manner can metabolomics studies lead to a better understanding of tumor biology, as exemplified by the recent identification of potential new potential disease biomarkers such as the metabolites that have been discussed in this review. Ultimately a better understanding of the specific drug targets could lead to a next generation of rationally-designed new drugs.

In conclusion, NMR is a reliable and highly reproducible experimental tool that has already proven to be extremely valuable in metabolomics studies. It allows for the detection of a broad range of polar soluble metabolites, while being nondestructive and keeping the sample in a close to native state, thus having a minimal impact on its metabolic composition. We suggest that NMR spectroscopy will continue to play an important role in metabolomics studies and in future cancer medicine.

## References

[1] Global Industry Analyst Inc. http://www.strategyr.com/Metabolomics_Market_Report.asp/.

[2] Ben Sellem D., Elbayed K., Neuville A., Moussallieh F.M., Lang-Averous G., Piotto M., Bellocq J.P., Namer I.J. (2011). Metabolomic characterization of ovarian epithelial carcinomas by hrmas-NMR spectroscopy. J. Oncol..

[3] Weljie A.M., Bondareva A., Zang P., Jirik F.R. (2011). ^1^H NMR metabolomics identification of markers of hypoxia-induced metabolic shifts in a breast cancer model system. J. Biomol. NMR.

[4] Bathe O.F., Shaykhutdinov R., Kopciuk K., Weljie A.M., McKay A., Sutherland F.R., Dixon E., Dunse N., Sotiropoulos D., Vogel H.J. (2011). Feasibility of identifying pancreatic cancer based on serum metabolomics. Cancer Epidemiol. Biomar. Prev..

[5] Tiziani S., Lopes L., Günther U.L. (2009). Early stage diagnosis of oral cancer using ^1^H NMR–based metabolomics. Neoplasia.

[6] Hasim A., Ma H., Mamtimin B., Abudula A., Niyaz M., Zhang L.W., Anwer J., Sheyhidin I. (2012). Revealing the metabonomic variation of EC using ^1^H-NMR spectroscopy and its association with the clinicopathological characteristics. Mol. Biol. Rep..

[7] Carrola J., Rocha C.M., Barros A.S., Gil A.M., Goodfellow B.K., Carreira I.M., Bernardo J., Gomes A., Sousa S., Carvalho L. (2011). Metabolic signatures of lung cancer in biofluids: NMR-based metabonomics of urine. J. Proteome Res..

[8] Teahan O., Bevan C.L., Waxman J., Keun H.C. (2011). Metabolic signatures of malignant progression in prostate epithelial cells. Int. J. Biochem. Cell. Biol..

[9] Cao M., Zhao L., Chen H., Xue W., Lin D. (2012). NMR-based metabolomic analysis of human bladder cancer. Anal. Sci..

[10] Chun E., Chan Y., Koon Koh P., Mal M., Yean Cheah P., Weng Eu K., Backshall A., Cavill R., Nicholson J.K., Keun H.C. (2009). Metabolic profiling of human colorectal cancer using high-resolution Magic angle spinning nuclear magnetic resonance (HR-MAS NMR) spectroscopy and gas chromatography mass spectrometry (GC/MS). J. Proteome Res..

[11] Farshidfar F., Weljie A.M., Kopciuk K., Buie W.D., Maclean A., Dixon E., Sutherland F.R., Molckovsky A., Vogel H.J., Bathe O.F. (2012). Serum metabolomic profile as a means to distinguish stage of colorectal cancer. Genome Med..

[12] Slupsky C.M., Steed H., Wells T.H., Dabbs K., Schepansky A., Capstick V., Faught W., Sawyer M.B. (2010). Urine metabolite analysis offers potential early diagnosis of ovarian and breast cancers. Clin. Cancer Res..

[13] Fong M.Y., McDunn J., Kakar S.S. (2011). Identification of metabolites in the normal ovary and their transformation in primary and metastatic ovarian cancer. PloS One.

[14] Warburg O. (1956). On the origin of cancer cells. Science.

[15] Locasale J.W., Grassian A.R., Melman T., Lyssiotis C.A., Mattaini K.R., Bass A.J., Heffron G., Metallo C.M., Muranen T., Sharfi H. (2011). Phosphoglycerate dehydrogenase diverts glycolytic flux and contributes to oncogenesis. Nat. Genet..

[16] Vander Heiden M.G., Locasale J.W., Swanson K.D., Sharfi H., Heffron G.J., Amador-Noguez D., Christofk H.R., Wagner G., Rabinowitz J.D., Asara J.M. (2010). Evidence for an alternative glycolytic pathway in rapidly proliferating cells. Science.

[17] (2006). Critical Path Opportunities Report. U.S Department of Health and Human Services Food and Drug Administration.

[18] Orphanos G., Kountourakis P. (2012). Targeting the HER2 receptor in metastatic breast cancer. Hematol. Oncol. Stem Cell Ther..

[19] Aiello M., Vella N., Cannavo C., Scalisi A., Spandidos D.A., Toffoli G., Buonadonna A., Libra M., Stivala F. (2011). Role of genetic polymorphisms and mutations in colorectal cancer therapy (Review). Mol. Med. Rep..

[20] National Cancer Institute. http://m.cancer.gov/topics/factsheets/targeted/.

[21] Lyng H., Sitter B., Bathen T.F., Jensen L.R., Sundfor K., Kristensen G.B., Gribbestad I.S. (2007). Metabolic mapping by use of high-resolution magic angle spinning ^1^H NMR spectroscopy for assessment of apoptosis in cervical carcinomas. BMC Cancer.

[22] Blankenberg F.G., Katsikis P.D., Storrs R.W., Beaulieu C., Spielman D., Chen J.Y., Naumovski L., Tait J.F. (1997). Quantitative analysis of apoptotic cell death using proton nuclear magnetic resonance spectroscopy. Blood.

[23] Lindon J.C., Keun H.C., Ebbels T.M.D., Pearce J.M.T., Holmes E., Nicholson J.K. (2005). The consortium for metabonomic toxicology (COMET): Aims, activities and achievements. Pharmacogenomics.

[24] Zhang A., Sun H., Wu X., Wang X. (2012). Urine metabolomics. Clin. Chim. Acta.

[25] Weljie A.M., Dowlatabadi R., Miller B.J., Vogel H.J., Jirik F.R. (2007). An inflammatory arthritis-associatedmetabolite biomarker pattern revealed by ^1^H NMR spectroscopy. J. Proteome Res..

[26] Daykin C.A., Foxall P.J., Connor S.C., Lindon J.C., Nicholson J.K. (2002). The comparison of plasma deproteinization methods for the detection of low-molecular-weight metabolites by ^1^H nuclear magnetic resonance spectroscopy. Anal. Biochem..

[27] Tiziani S., Emwas A.H., Lodi A., Ludwig C., Bunce C.M., Viant M.R., Gunther U.L. (2008). Optimized metabolite extraction from blood serum for ^1^H nuclear magnetic resonance spectroscopy. Anal. Biochem..

[28] Soininen P., Kangas A.J., Würtz P., Tukiainen T., Tynkkynen T., Laatikainen R., Järvelin M-J., Kähönen M., Lehtimäki T., Viikari J. (2009). High-throughput serum NMR metabonomics for cost-effective holistic studies on systemic metabolism. Analyst.

[29] Schicho R., Shaykhutdinov R., Ngo J., Nazyrova A., Schneider C., Panaccione R., Kaplan G.G., Vogel H.J., Storr M. (2012). Quantitative metabolomic profiling of serum, plasma, and urine by ^1^H NMR spectroscopy discriminates between patients with inflammatory bowel disease and healthy individuals. J. Proteome Res..

[30] Weljie A.M., Newton J., Mercier P., Carlson E., Slupsky C.M. (2006). Targeted Profiling: quantitative analysis of ^1^H NMR metabolomics data. Anal. Chem..

[31] Nicholson J.K., Foxhall P.J.D. (1995). 750 MHz ^1^H and ^1^H-^13^C NMR spectroscopy of human blood plasma. Anal. Chem..

[32] Van Q.N., Chmurny G.N., Veenstra T.D. (2003). The depletion of protein signals in metabonomics analysis with the WET–CPMG pulse sequence. Biochem. Biophys. Res. Commun..

[33] Ludwig C., Viant M.R. (2010). Two-dimensional J-resolved NMR spectroscopy: review of a key methodology in the metabolomics toolbox. Phytochem. Anal.: PCA.

[34] Fonville J.M., Maher A.D., Coen M., Holmes E., Lindon J.C., Nicholson J.K. (2010). Evaluation of full-resolution J-resolved ^1^H NMR projections of biofluids for metabonomics information retrieval and biomarker identification. Anal. Chem..

[35] Viant M.R. (2003). Improved methods for the acquisition and interpretation of NMR metabolomic data. Biochem. Biophys. Res. Commun..

[36] Wang Y., Bollard M.E., Keun H., Antti H., Beckonert O., Ebbels T.M., Lindon J.C., Holmes E., Tang H., Nicholson J.K. (2003). Spectral editing and pattern recognition methods applied to high-resolution magic-angle spinning ^1^H nuclear magnetic resonance spectroscopy of liver tissues. Anal. Biochem..

[37] Bertram H.C., Eggers N., Eller N. (2009). Potential of human saliva for nuclear magnetic resonance-based metabolomics and for health-related biomarker identification. Anal. Chem..

[38] Monleon D., Morales J.M., Barrasa A., Lopez J.A., Vazquez C., Celda B. (2009). Metabolite profiling of fecal water extracts from human colorectal cancer. NMR Biomed..

[39] Wishart D.S., Lewis M.J., Morrissey J.A., Flegel M.D., Jeroncic K., Xiong Y., Cheng D., Eisner R., Gautam B., Tzur D. (2008). The human cerebrospinal fluid metabolome. J. Chromatogr. B Analyt. Technol. Biomed. Life Sci..

[40] Hügle T., Kovats H., Heijnen I.A., Daikeler T., Baisch U., Hicks J.M., Valderrabano V. (2012). Synovial fluid metabolomics in different forms of arthritis assessed by nuclear magnetic resonance spectroscopy. Clin. Exp. Rheumatol..

[41] Moestue S., Sitter B., Frost Bathen T., Tessem M-B., Gribbestad I.S. (2011). HR MAS MR spectroscopy in metabolic characterization of cancer. Curr. Top. Med. Chem..

[42] Ferentz A.E., Wagner G. (2000). NMR spectroscopy : A multifaceted approach to macromolecular structure. Q Rev. Biophys..

[43] Bax A., Grishaev A. (2005). Weak alignment NMR: A hawk-eyed view of biomolecular structure. Curr. Opin. Struct. Biol..

[44] Tugarinov V., Hwang P.M., Kay L.E. (2004). Nuclear magnetic resonance spectroscopy of high-molecular-weight proteins. Annu. Rev. Biochem..

[45] Pellecchia M., Sem D.S., Wuthrich K. (2002). NMR in drug discovery. Nat. Rev. Drug Discov..

[46] Hajduk P.J., Meadows R.P., Fesik S.W. (1999). NMR-based screening in drug discovery. Q Rev. Biophys..

[47] Jourdan C., Petersen A.K., Gieger C., Doring A., Illig T., Wang-Sattler R., Meisinger C., Peters A., Adamski J., Prehn C. (2012). Body fat free mass is associated with the serum metabolite profile in a population-based study. PloS One.

[48] Putri S.P., Nakayama Y., Matsuda F., Uchikata T., Kobayashi S., Matsubara A., Fukusaki E. (2013). Current metabolomics: Practical applications. J. Biosci. Bioeng..

[49] Koek M.M., Jellema R.H., van der Greef J., Tas A.C., Hankemeier T. (2011). Quantitative metabolomics based on gas chromatography mass spectrometry: Status and perspectives. Metabolomics.

[50] Griffin J.L., Shockcor J.P. (2004). Metabolic profiles of cancer cells. Nat. Rev. Cancer.

[51] Nicholson J.K., Conelly J., Lindon J.C., Holmes E. (2002). Metabonomics: A platform for studying drug toxicity and gene function. Nat. Rev. Drug Discov..

[52] Gowda G.A., Zhang S., Gu H., Asiago V., Shanaiah N., Raftery D. (2008). Metabolomics-based methods for early disease diagnostics. Exp. Rev. Mol. Diagn.

[53] Bathen T.F., Sitter B., Sjobakk T.E., Tessem M.B., Gribbestad I.S. (2010). Magnetic resonance metabolomics of intact tissue: A biotechnological tool in cancer diagnostics and treatment evaluation. Cancer Res..

[54] Dunn W.B., Broadhurst D.I., Atherton H.J., Goodacre R., Griffin J.L. (2011). Systems level studies of mammalian metabolomes: the roles of mass spectrometry and nuclear magnetic resonance spectroscopy. Chem. Soc. Rev..

[55] Martin P., Oliver S., Kennedy S.J., Partridge E., Hutchison M., Clarke D., Giles P. (2012). Pharmacokinetics of vandetanib: Three phase I studies in healthy subjects. Clin. Ther..

[56] Cohen M.H., Hirschfeld S., Flamm Honig S., Ibrahim A., Johnson J.R., O’Leary J.J., White R.M., Williams G.A., Pazdur R. (2001). Drug approval summaries: arsenic trioxide, tamoxifen citrate, anastrazole, paclitaxel, bexarotene. Oncologist.

[57] Schnackenberg L., Beger R.D., Dragan Y. (2005). NMR-based metabonomic evaluation of livers from rats chronically treated with tamoxifen, mestranol, and phenobarbital. Metabolomics.

[58] Tenori L., Oakman C., Claudino W.M., Bernini P., Cappadona S., Nepi S., Biganzoli L., Arbushites M.C., Luchinat C., Bertini I. (2012). Exploration of serum metabolomic profiles and outcomes in women with metastatic breast cancer: a pilot study. Mol. Oncol..

[59] National Cancer Institute. http://www.cancer.gov/newscenter/qa/2001/gleevecqa/.

[60] Druker B.J., Guillot. F., O’Brien S.G., Gathmann I., Kantarjian H., Gattermann N., Deininger M.W.N., Silver R.T., Goldman J.M., Stone R.M. (2006). A five-year follow-UP of patients receiving imatinib for chronic myeloid leukemia. N Engl. J. Med..

[61] Hochhaus A., O'Brien S.G., Guilhot F., Druker B.J., Branford S., Foroni L., Goldman J.M., Muller M.C., Radich J.P., Rudoltz M. (2009). Six-year follow-up of patients receiving imatinib for the first-line treatment of chronic myeloid leukemia. Leukemia.

[62] Saito S., Nakata K., Kajiura S., Ando T., Hosokawa A., Sugiyama T. (2013). Long-term follow-up outcome of imatinib mesylate treatment for recurrent and unresectable gastrointestinal stromal tumors. Digestion.

[63] Dewar B.J., Keshari K., Jeffries R., Dzeja P., Graves L.M., Macdonald J.M. (2010). Metabolic assessment of a novel chronic myelogenous leukemic cell line and an imatinib resistant subline by ^1^H NMR spectroscopy. Metabolomics.

[64] Dengler M.A., Staiger A.M., Gutekunst M., Hofmann U., Doszczak M., Scheurich P., Schwab M., Aulitzky W.E., van der Kuip H. (2011). Oncogenic stress induced by acute hyper-activation of Bcr-Abl leads to cell death upon induction of excessive aerobic glycolysis. PloS One.

[65] Spratlin J.L., Pitts T.M., Kulikowski G.N., Morelli M.P., Tentler J.J., Serkova N.J., Eckhardt S.G. (2011). Synergistic activity of histone deacetylase and proteasome inhibition against pancreatic and hepatocellular cancer cell lines. Anticancer Res..

[66] National Center for Advancing Translational Sciences. http://www.ncats.nih.gov/research/reengineering/process.html/.

[67] Cavill R., Keun H.C., Holmes E., Lindon J.C., Nicholson J.K., Ebbels T.M. (2009). Genetic algorithmsfor simultaneous variable and sample selection in metabonomics. Bioinformatics.

[68] Coen M., Holmes E., Lindon J.C., Nicholson J.K. (2008). NMR-based metabolic profiling and metabonomic approaches to problems in molecular toxicology. Chem. Res. Toxicol..

[69] Verstappen C.C.P., Heimans J.J., Hoekman K., Postma T.J. (2003). Neurotoxic complications of chemotherapy in patients with cancer. Drugs.

[70] Lyman G.H. (2009). Impact of chemotherapy dose intensity on cancer patient outcomes. J. Natl. Compr. Cancer Network.

[71] Sorg B.L., Hull W.E., Kliem H.C., Mier W., Wiessler M. (2005). Synthesis and NMR characterization of hydroxyurea and mesylglycol glycoconjugates as drug candidates for targeted cancer chemotherapy. Carbohydr Res..

[72] Naser-Hijazi B., Berger M.R., Schmähl D., Schlag P., Hull W.E. (1991). Locoregional administration of 5-fluoro-2'-deoxyuridine (FdUrd) in Novikoff hepatoma in the rat: effects of dose and infusion time on tumor growth and on FdUrd metabolite levels in tumor tissue as determined by 19F-NMR spectroscopy. J. Cancer Res. Clin..

[73] Backshall A., Sharma R., Clarke S.J., Keun H.C. (2011). Pharmacometabonomic profiling as a predictor of toxicity in patients with inoperable colorectal cancer treated with capecitabine. Clin. Cancer Res..

[74] Adamski J., Suhre K. (2013). Metabolomics platforms for genome wide association studies-linking the genome to the metabolome. Curr. Opin. Biotechnol..

[75] Wang Q., Jiang Y., Wu C., Zhao J., Yu S., Yuan B., Yan X., Liao M. (2006). Study of a novel indolin-2-ketone compound Z24 induced hepatotoxicity by NMR-spectroscopy-based metabonomics of rat urine, blood plasma, and liver extracts. Toxicol. Appl. Pharmacol..

[76] Wei S., Liu L., Zhang J., Bowers J., Gowda G.A., Seeger H., Fehm T., Neubauer H.J., Vogel U., Clare S.E., Raftery D. (2012). Metabolomics approach for predicting response to neoadjuvant chemotherapy for breast cancer. Mol. Oncol..

[77] El-Deredy W., Ashmore. S.M., Branston N.M., Darling J.L., Williams S.R., Thomas D.G.T. (1997). Pretreatment prediction of the chemotherapeutic response of human glioma cell cultures using nuclear magnetic resonance spectroscopy and artifical neural networks. Cancer Res..

[78] Triba M.N., Starzec A., Bouchemal N., Guenin E., Perret G.Y., Le Moyec L. (2010). Metabolomic profiling with NMR discriminates between biphosphonate and doxorubicin effects on B16 melanoma cells. NMR Biomed..

[79] Bayet-Robert M., Loiseau D., Rio P., Demidem A., Barthomeuf C., Stepien G., Morvan D. (2010). Quantitative two-dimensional HRMAS ^1^H-NMR spectroscopy-based metabolite profiling of human cancer cell lines and response to chemotherapy. Magn. Reson. Med..

[80] Tiziani S., Lodi A., Khanim F.L., Viant M.R., Bunce C.M., Gunther U.L. (2009). Metabolomic profiling of drug responses in acute myeloid leukaemia cell lines. PloS One.

[81] Mailloux J.M., Bériault R., Lemire J., Singh R., Chénier D.R., Hamel R.H., Appanna V.D. (2007). The tricarboxylic acid cycle, an ancient metabolic network with a novel twist. PloS One.

[82] Sonneveld P., Schmidt-Wolf I.G., van der Holt B., El Jarari L., Bertsch U., Salwender H., Zweegman S., Vellenga E., Broyl A., Blau I.W. (2012). Bortezomib induction and maintenance treatment in patients with newly diagnosed multiple myeloma: results of the randomized phase III HOVON-65/ GMMG-HD4 trial. J. Clin. Oncol..

[83] Giaccone G., Rajan A., Berman A., Kelly R.J., Szabo E., Lopez-Chavez A., Trepel J., Lee M.J., Cao L., Espinoza-Delgado I. (2011). Phase II study of belinostat in patients with recurrent or refractory advanced thymic epithelial tumors. J. Clin. Oncol..

[84] US National Institutes of Health. http://clinicaltrials.gov/ct2/show/NCT00431340?term=belinostat&rank=16/.

[85] Pan X., Wilson M., Mirbahai L., McConville C., Arvanitis T.N., Griffin J.L., Kauppinen R.A., Peet A.C. (2011). In vitro metabonomic study detects increases in UDP-GlcNAc and UDP-GalNAc, as early phase markers of cisplatin treatment response in brain tumor cells. J. Proteome Res..

[86] Gu Y., Mi W., Ge Y., Liu H., Fan Q., Han C., Yang J., Han F., Lu X., Yu W. (2010). GlcNAcylation plays an essential role in breast cancer metastasis. Cancer Res..

[87] Brooks S.A., Carter T.M., Bennett E.P., Clausen H., Mandel U. (2007). Immunolocalisation of members of the polypeptide N-acetylgalactosaminyl transferase (ppGalNAc-T) family is consistent with biologically relevant altered cell surface glycosylation in breast cancer. Acta Histochem..

[88] Bayet-Robert M., Morvan D., Chollet P., Barthomeuf C. (2010). Pharmacometabolomics of docetaxel-treated human MCF7 breast cancer cells provides evidence of varying cellular responses at high and low doses. Breast Cancer Res. Treat..

[89] Bayet-Robert M., Lim S., Barthomeuf C., Morvan D. (2010). Biochemical disorders induced by cytotoxic marine natural products in breast cancer cells as revealed by proton NMR spectroscopy-based metabolomics. Biochem. Pharmacol..

[90] Zhang A., Sun H., Wang Z., Sun W., Wang P., Wang X. (2010). Metabolomics: Towards understanding traditional Chinese medicine. Planta Med..

[91] Halouska S., Fenton R.J., Barletta R.G., Powers R. (2012). Predicting the *in vivo* mechanism of action for drug leads using NMR metabolomics. ACS Chem. Biol..

[92] Bertini I., Cacciatore S., Jensen B.V., Schou J.V., Johansen J.S., Kruhoffer M., Luchinat C., Nielsen D.L., Turano P. (2012). Metabolomic NMR fingerprinting to identify and predict survival of patients with metastatic colorectal cancer. Cancer Res..

[93] Lodi A., Tiziani S., Khanim F.L., Gunther U.L., Viant M.R., Morgan G.J., Bunce C.M., Drayson M.T. (2013). Proton NMR-based metabolite analyses of archived serial paired serum and urine samples from myeloma patients at different stages of disease activity identifies acetylcarnitine as a novel marker of active disease. PloS One.

[94] US Food and Drug Administration. http://www.fda.gov/Drugs/DrugSafety/PostmarketDrugSafetyInformationforPatientsandProviders/ucm193900.html/.

[95] Borgan E., Lindholm E.M., Moestue S., Maelandsmo G.M., Lingjaerde O.C., Gribbestad I.S., Borresen-Dale A.L., Engebraaten O., Sorlie T. (2012). Subtype-specific response to bevacizumab is reflected in the metabolome and transcriptome of breast cancer xenografts. Mol. Oncol..

[96] Abaffy T., Moller M., Riemer D.D., Milikowski C., Defazio R.A. (2011). A case report—Volatile metabolomic signature of malignant melanoma using matching skin as a control. J. Cancer Sci. Ther..

[97] Vriens D., de Geus-Oei L.F., Heerschap A., van Laarhoven H.W., Oyen W.J. (2011). Vascular and metabolic response to bevacizumab-containing regimens in two patients with colorectal liver metastases measured by dynamic contrast-enhanced MRI and dynamic 18F-FDG-PET. Clin. Colorectal. Cancer.

